# Violence Exposure and Mental Health of College Students in the United States

**DOI:** 10.3390/bs8060053

**Published:** 2018-05-24

**Authors:** Shervin Assari, Maryam Moghani Lankarani

**Affiliations:** 1 Department of Psychiatry, University of Michigan, Ann Arbor, MI 48109, USA; lankaranii@yahoo.com; 2 Center for Research on Ethnicity, Culture and Health, School of Public Health, University of Michigan, Ann Arbor, MI 48109, USA

**Keywords:** abuse, violence, sexual abuse, depression, anxiety, substance use, suicidal behaviors

## Abstract

**Background**: Despite the well-established link between exposure to violence and mental health problems, less is known about this association among college students. The current study aimed to investigate the association between history of exposure to violence and mental health of American college students. **Methods**: Healthy Mind Study (HMS, 2016–2017) is a national online survey of 41,898 adult college students. The independent variable was lifetime history of exposure to violence (psychological, physical, and sexual). The dependent variables were anxiety, depression, and suicidality. Race, age, gender, sexual orientation, parental education, financial stress, transfer status, enrollment status, and graduate status were covariates. Linear and logistic regression models were used for data analysis. **Results**: History of exposure to violence was associated with all three aspects of poor mental health, namely general anxiety, depression, and suicidality. These associations were independent of covariates and type of abuse. **Conclusions**: There is a need to address various mental health needs of college students who have experienced various forms of violence. College students who screen positive for history of violence exposure should be evaluated for anxiety, depression, and suicidal behaviors.

## 1. Background

For a wide range of reasons, college students are at an increased risk of mental health problems [1]. Transition to college is a stressful challenge for many students, which requires effective adjustment and coping [2]. Attending college is associated with considerable stress as a direct result of academic pressure [3]. In addition, some students, experience other sources of stress such as separation from family, emotional intimacy, individual responsibility, and work-related stress for the first time [4]. Many college students experience multiple sources of stress which increases their risk of mental health problems [4]. Given the unique stressors in the lives of college students, there is a need to understand the specific risk factors that affect mental health of this population [1].

In a review of mental health among college students, Pedrelli et al., listed anxiety, depression, and suicidality as the top three mental health problems that require the most attention on college campuses [1]. Anxiety, which may present as generalized anxiety disorders (GAD), social phobia, or post-traumatic stress disorder (PTSD), is the most prevalent psychiatric problem among college students, affecting about 12% of all college students [5,6]. Depression is possibly the second most common mental health problem among college students with a prevalence of about 9% [5,7]. Suicidal behaviors, while they can co-occur with other mental health problems, are significant problems among college students and the third leading cause of death among young adults [8]. Among students, about 7% report suicidal ideation, 2% report a suicidal plan, and 0.5% report a suicide attempt in the past year [9]. Given that anxiety, depression, and suicidal behaviors tend to co-occur [10,11,12,13,14,15,16,17], some students suffer from more than one of these mental health problems [18,19]. Still, many students do not seek treatment [20,21]. As a result, it is critical to understand risk factors that may help with the screening of mental health needs of college students.

Several studies have investigated a wide range of social factors that may increase risk of poor mental health, including suicidal behaviors in college students [22]. Gender [23], race [24,25], financial hardship [23,26], sexual orientation minority status (non-heterosexual orientation) [27], transfer status [28], and parental education [29,30] have been reported as some of the risk factors for poor mental health of college students.

A unique risk factor for poor mental health that requires additional research is violence victimization [6,16,30,31,32,33,34,35,36]. In one study, violence victimization was the strongest predictor of suicidality of college students [37]. Most of the literature on exposure to violence is either in the community, clinical, or correctional settings [5,38,39,40,41,42], with very few studies available on mental health correlates of history of exposure to violence on college campuses. Individuals experience various forms of violence, which include physical, psychological, and sexual abuse [43,44]. Regardless of type of violence [44], victims of violence are at a higher risk of poor psychological outcomes [7,44,45]. Literature has shown that psychological, physical, and sexual abuse tend to co-occur [8,9,46,47,48,49] and victims of one type are more likely to experience other types [49]. For instance, individuals who are exposed to psychological abuse are also more likely experience sexual and physical violence, particularly in women [48]. Various forms of abuse have common [50] and unique [47,51,52] social, behavioral, and psychological risk factors. Minority status and low socioeconomic status [53,54,55,56], female gender [57], low and high ends of the age spectrum [58,59], substance use [60,61], depression [62,63], anxiety [62,63], and relation problems [45] all increase risk of violence victimization. Most of this literature, however, is from studies with local data that does not result in generalizable estimates that can inform public policies. Another issue lack of comparability as some of these studies are from countries outside the US, which their results may not be similar to US.

Outside the context of college campuses, a mixed literature exists on the association between violence and poor mental health [64,65,66] as only some [57,58,59], but not all [67,68,69,70], studies have shown such an association. Using a national data [71,72], the current study investigated the association between history of exposure to violence and psychological health of American college students. We also tested the same association for each type of violence (psychological, physical, and sexual).

## 2. Methods

### 2.1. Design and Setting

With a cross-sectional design, this study used data from the Healthy Mind Study (HMS), an online mental health survey of American college students. HMS is an annual web-based survey that examines mental well-being of undergraduate and graduate students in the United States. The study collects data on sociodemographic factors, mental health status, stigma, substance use, and service utilization [71,72]. Since its launch in 2007, HMS has collected data from 150 colleges and universities, with over 175,000 survey respondents. As a web-based survey, HMS uses three standard survey modules on all participating campuses: demographics, mental health, and service utilization. The HMS is conducted on an annual basis. For this particular study, we used year 2016–2017 data.

### 2.2. Ethics

The HMS fully protects the privacy of its participants and confidentiality of their data. The HMS protocol was approved by the University of Michigan Health Sciences and Behavioral Sciences Institutional Review Board (IRB). The study was covered by a Certificate of Confidentiality from the National Institutes of Health (NIH). All participants provided informed consent. The survey was anonymous.

### 2.3. Sampling and Participants

HMS is a web-based survey. Students are invited and reminded to participate in the survey via emails, which are timed to avoid, if at all possible, the first two weeks of the term, the last week of the term, and any major holidays. The data collection protocol begins with an email invitation, and non-responders are contacted up to three times by email reminders spaced by 2–4 days each. Reminders are only sent to those who have not yet completed the survey. Each communication contains a URL that students use to gain access to the survey.

Each participating school provides the HMS team with a randomly selected sample of currently enrolled students over the age of 18. In most cases, large schools provide a random sample of 4000 students, while smaller schools use census (all students). Schools with graduate students typically include both undergraduates and graduate students in the sample. The study does not have specific exclusion criteria.

### 2.4. Measures

#### 2.4.1. Independent Variable

*Exposure to violence.* The independent variable in this study was history of exposure to lifetime violence measured using three items: psychological, physical, and sexual violence. Items included: (1) *“Over the past 12 months, were you called names, yelled at, humiliated, judged, threatened, coerced, or controlled by another person?”*; (2) *“Over the past 12 months, were you kicked, slapped, punched or otherwise physically mistreated by another person?”*; and (3) *“In the past 12 months, has anyone had unwanted sexual contact with you?”* Responses were yes (1) and no (0). We operationalized our violence exposure as a dichotomous variable, which reflected any exposure, regardless of its type. Thus, our measure of “any violence” reflected individuals who suffered emotional, physical, or sexual violence, regardless of the type. A subset of these individuals had experienced multiple forms of violence simultaneously.

#### 2.4.2. Dependent Variables

Dependent variables were three mental health outcomes: depression, anxiety, and suicidality.

*Depression.* Depression was measured using the Patient Health Questionnaire (PHQ-9). In line with the Composite International Diagnostic Interview Short-Form [73], participants were asked to think about the 2-week period with the highest symptom levels: “Think about the two-week period in the past year when you experienced the two problems below the most frequently. During that period, how often were you bothered by these problems?” We then listed the PHQ items such as “Little interest or pleasure in doing things” and “Feeling down, depressed, or hopeless”. Item responses used a four-level category ranging from 0 (none) to 3 (nearly every day) points per item, where a higher score reflected more symptom frequency [74].

*Anxiety.* General anxiety was measured using the 7-item Generalized Anxiety Disorder (GAD-7) scale [75], a self-report questionnaire designed to identify probable cases of generalized anxiety disorder. GAD-7 measures symptom severity over the past 2 weeks [75,76]. GAD-7 is designed based on the DSM-IV diagnostic criteria. GAD-7 asks participants how frequently, during the last 2 weeks, have they experienced seven core symptoms of generalized anxiety disorder. Item responses range from (0) “not at all,” to (3) “nearly every day”. A total score is calculated, with a higher score reflecting higher symptoms. This measure has shown high reliability, construct validity, and factorial validity in the general population and clinical sample [74].

*Suicidal Behaviors*. The following three aspects of suicidal behaviors were measured: (1) suicidal ideation, (2) suicidal plans, and (3) suicidal attempts. A yes/no question was used to measure each aspect of suicidality. These items were taken from the National Comorbidity Survey (NCS). The questions were “In the past year, did you ever seriously think about committing suicide?” “In the past year, did you make a plan for committing suicide?” and “In the past year, did you attempt suicide?” The two last questions were only asked if the responses to the 1st item was “yes” [77]. We operationalized our suicidality as a dichotomous variable, which reflects any suicidal behaviors, regardless of their type.

#### 2.4.3. Confounders

The study evaluated the following variables: race and ethnicity, parental socioeconomic status (SES), gender, age, sexual orientation, parental education, history of exposure to violence, and mental health (depression, general anxiety, and suicidality).

*Race and ethnicity.* Race and ethnicity were measured as a dichotomous variable (1 White, 0 others). Others included (1) African American/Black; (2) American Indian or Alaskan Native; (3) Asian American/Asian; (4) Hispanic/Latino; (5) Native Hawaiian or Pacific Islander; (6) Middle Eastern, Arab, or Arab American; and (7) others. We operationalized race as White vs non-White (reference group).

*Demographic variables.* Age and gender were measured as demographic characteristics. Age was a continuous measure. Gender was a dichotomous variable with male as the reference group.

*Sexual orientation.* Sexual orientation was measured using the item “How would you describe your sexual orientation?”. Responses include (1) Heterosexual; (2) Lesbian; (3) Gay; (4) Bisexual; (5) Queer; (6) Questioning; (7) Others. We operationalized sexual orientation as a dichotomous variable (1 heterosexual, 0 others).

### 2.5. Statistical Power Calculation

Post-hoc Statistical Power Calculator for Multiple Regression. Observed statistical power of 0.99 was achieved with 15 predictors, 5% observed R2, probability level of 0.001, and sample size of 40,000.

### 2.6. Data Analysis

We used SPSS 24.00 (SPSS Inc., Chicago, IL, USA) for data analysis. For descriptive purposes, we reported frequency tables (%) and means (SD). Pearson correlation test wad calculated for bivariate analysis. We ran multiple logistic and linear regression models, depending on the type of outcome. The independent variable in this study was history of exposure to violence measured using three items (psychological, physical, and sexual violence) in lifetime. The dependent variable for this study was mental health measured using various measures (Patient Health Questionnaire, general anxiety, suicidality). Age, gender, sexual orientation, SES (parental education and financial stress), and enrollment status were covariates. We did not apply specific model selection procedures to finalize our models. All models had similar covariates. We replicated the results for each type of violence.

From our logistic regression models, we reported Odds Ratio (OR), 95% CI, and *p* values. From our linear regression models, adjusted unstandardized b (regression coefficients), associated 95% confidence interval (CI), and *p* values were reported. *p* less than 0.05 were considered statistically significant.

## 3. Results

Descriptive statistics are shown in Table 1. 15.74% of the students reported history of violence victimization.

Table 2 summarizes the results of four linear regression models with any violence victimization, emotional abuse, physical abuse and sexual abuse as the independent variables and symptoms of anxiety as the outcome. History of exposure to all types of violence were positively associated with GAD score, independent of covariates. Positive history of violence exposure was associated with 2.74 score increase in GAD score. This increase was 2.80, 2.81, and 2.54 for emotional, physical, and sexual abuse (Table 2).

Table 3 provides a summary of the results of a linear regression model in which violence victimization was the independent variable and symptoms of depression was the outcome. History of exposure to violence was positively associated with depression, net of covariates. Positive history of violence exposure was associated with 3.09 score increase in PHQ score. This increase was 3.14, 3.05, and 3.05 for emotional, physical, and sexual abuse (Table 3).

Table 4 summarizes the results of a logistic regression model with violence victimization as the independent variable and suicidality as the outcome. History of exposure to violence was positively associated with suicidality above and beyond covariates. Positive history of violence exposure was associated with 2.98 higher odds of suicidal ideation. The increased odds of suicidal behaviors were 2.90, 2.81, and 2.74 for emotional, physical, and sexual abuse (Table 4).

## 4. Discussion

This study had two main findings. First, the study showed an association between history of exposure to violence and various aspects of poor psychological outcomes including anxiety, depression, and suicidal behaviors. Second, these associations were independent of demographic and socioeconomic factors, and consistent across trauma types.

The first result of the current study was supported by findings of previous research which have shown a positive link between history of abuse, neglect, interpersonal violence, and other types of violence victimization with poor mental health [6,32,78] such as anxiety [79] depression [10,62,63], and suicidal behaviors [16,33,34,35]. Perpetration and victimization of violence are linked to depression and anxiety in both males and females [62,63,80,81,82,83]. In a longitudinal study of 2337 college students in Belgium, previous exposure to physical abuse and also dating violence prior to the age of 17 were the strongest predictors for first-onset suicidal ideation (ORs = 4.23–12.25) and plans (ORs = 6.57–17.58) [37].

The second result of this study was different from what existing literature suggests. Some studies have shown that correlates of violence depend on the type of violence [47]. In particular, some studies have shown that sexual violence has a stronger effect on mental health, and physical violence has a greater consequence than emotional abuse [78,84,85,86].

Only a few studies have ever linked exposure to violence and poor mental health in college students [16,33,34,35,36,87]. A study used data from the National Epidemiologic Study on Alcohol and Related Conditions (NESARC) and examined associations between the prevalence of seven violent behaviors and 19 psychiatric disorder diagnoses tapping mood, anxiety, personality, and substance use disorders. Authors asked respondents about 33 different antisocial behaviors. According to that study, which included a national sample of 3929 American college students, presence of psychiatric disorders was associated with an increased risk of violent behavior among college students [87].

In our study, history of violence victimization was a universal risk factor for all three mental health outcomes investigated. The mechanisms for the effects of violence victimization on anxiety, depression, and suicidality may be universal or specific to each outcome. Multiple mechanisms explain why victims of violence are at a higher risk of several poor psychological outcomes [7,44,45]. First and foremost, violence victimization is a form of stress, and all types of stress increase risk of psychopathology at a population level [88]. This is supported by studies that have shown HPA axis dys-regulation is a mechanism that links violence victimization to psychopathology [89]. Stressful events such as abuse are particularly linked to anxiety disorders such as post-traumatic stress disorder [90]. Deterioration of emotion regulation is a common pathway that can link victimization and other stressors to a wide range of mental health problems [91]. All these negative cognitions about self (self-blame) and others may increase risk of depression and suicide [92]. Violence victimization may also have unique features that are different from other types of stressful conditions. Violence victims may be at higher risk of loneliness and social withdrawal [93], which are well known predictors of psychopathology and suicidality [94]. Another unique risk factor for psychopathology is rumination, which increases vulnerability to stress [95]. Finally, poor SES, gender, minority status, and several other third factors may cause both violence victimization and poor mental health. Some examples of these third variables include social disorder, economic stress, disrupted family, poor parenting, and unsafe neighborhoods [96,97].

This study controlled for a wide range of social factors such as race, financial difficulty, parental education, and sexual minority status. Minorities, and low socio-economic status (SES) individuals [53,54,55,56] are at higher risk of poor mental health, impulse control, and violence [98,99,100]. As socioeconomic factors increase risk of mental health problems and violence victimization, the association between them may be causation or selection.

### 4.1. Implications

Our study has some policy and clinical implications. There is a need for addressing the various mental health needs of college students who experienced violence. There is a need for attention to mental health needs of college students who have a history of violence victimization in their lifetime. In the presence of history of violence exposure, there is a need for screening and treatment programs for college students. As minority status, low SES, violence exposure, and poor mental health tend to co-occur, there is a need for combined programs that jointly address prevention of violence and mental health problems, especially among minority and low SES students.

The current study advocates for a trauma-informed approach to college mental health. The results can inform trauma-informed interventions that consider history of trauma as a core contextual aspect of mental health programs [101]. For instance, Trauma, Addiction, Mental Health, and Recovery (TAMAR) is designed for individuals with comorbid history of trauma and mental health problems [102]. Trauma Affect Regulation: Guide for Education and Therapy (TARGET) is another approach that helps the trauma survivor to de-escalate and regulate extreme emotions, manage intrusive trauma memories experienced in daily life, and restore the capacity for information processing and memory. All these models have capacity to be used for co-occurring trauma and mental disorders [103]. Such approaches consider trauma as a context in which mental health problems occur, and integrate cognitive, behavioral, interpersonal, and case management for increasing the chance of successful recovery [101]. These programs and this approach are being advocated by SAMHSA [101].

One very successful example was the introduction and implementation of suicide prevention programs after 21 October 2004, when the *Garrett Lee Smith Memorial Act* (GLSMA) was signed. The law supported three important programs: (1) The Suicide Prevention Resource Center; (2) Youth Suicide Intervention and Prevention Strategy Grants to States and Tribes; and (3) Mental Health and Substance Use Disorder Services and Outreach on Campus. This act provided states funding to expand their diagnosis and treatment services that collectively reduced on campus suicide [104]. Other programs should enable colleges and universities to promote college students’ mental health ad to prevent youth suicide. Since 2005, 175 institutions of higher education have implemented such programs. Local programs that implement suicide preventions receive federal monitoring and oversight coming from Substance Abuse and Mental Health Services Administration (SAMHSA) [104,105,106]. Various colleges, however, vary in the resources they specify for mental health promotion and suicide prevention.

### 4.2. Limitations

The current study had a few methodological limitations. Firstly, this was a cross-sectional study. As a result, the results should only be interpreted as association not causation. The measurement was also another limitation in this study. Low reliability of self-reported measurements of victimization is a threat to validity of the study results. Similar to other individuals, college students may have a tendency to underreport their exposure to violence and suicidal behaviors. This might be partially due to stigma, concerns about confidentiality, and social desirability. Such social desirability may depend on social factors such as gender, SES, minority status, and age. This study also did not differentiate among different levels of victimization (e.g., minor and major). We also did not focus on nuances based on race/ethnicity and sexual orientation, which were defined broadly. Future research may use multiple informants as well as archive and health care data to measure history of violence, suicide, and drug use.

### 4.3. Future Research

Race and sexual orientation were dichotomized in the current study. Future research should test differences in these associations by racial ethnic group, and also by sexual orientation. Additional research is needed to better understand the most effective policies and programs that promote mental health on campus. It is still unknown how sub-populations differ in the associations between SES, violence victimization, and poor mental health. In addition, there is a need to test the efficacy of programs and interventions that can potentially improve mental health of college students on a large scale. Prevention of violence may be one of many strategies for mental health promotion of college students. Future research should find more reliable tools that can collect most relevant data that can inform tailored treatment. Research should also assess the role of college variation in the resources available on campus for mental health support. Future studies should also use a longitudinal design with multiple observations to address temporal ambiguity issues. Other researchers may also explore nuances such as type of violence victimization and type of suicidal behavior.

## 5. Conclusions

To conclude, a history of exposure to violence was associated with various aspects of poor mental health including anxiety, depression, and suicidality. This association was independent of covariates and stayed stable for types of violence and mental health outcomes. College students who have a history of exposure to various types of violence require mental health screening. Given the limitations such as a relatively homogeneous sample, a cross-sectional design, self-reported measurements, and low levels of self-reported victimization, future research is needed.

## Figures and Tables

**Table 1:** Descriptive characteristics in the participants.

	*n*	%
Gender		
Male	12,982	34.68
Female	24,456	65.32
Heterosexual		
No	6174	16.54
Yes	31,158	83.46
Race		
Others	10,168	27.13
White	27,305	72.87
Enrollment		
Full-time student	33,617	89.81
Part-time student	3528	9.43
Other (please specify)	285	0.76
Transfer		
Transferred from a community or junior college	4472	17.09
Transferred from a 4-year college or university	2183	8.34
No	19,515	74.57
Violence Victimization		
No	25,237	84.26
Yes	4716	15.74

**Table 2:** Summary of linear regressions for the association between violence victimization and symptoms of general anxiety.

	b	95% CI	b	95% CI	b	95% CI	b	95% CI
	Any		Emotional		Physical		Sexual	
Age	−0.05 ***	−0.06–0.03	−0.05 ***	−0.06–0.03	−0.05 ***	−0.07–0.03	−0.05 ***	−0.07–0.03
Female	1.24 ***	1.09–1.39	1.25 ***	1.10–1.40	1.44 ***	1.28–1.59	1.40 ***	1.25–1.56
Heterosexual	−1.93 ***	−2.12–1.74	−1.92 ***	−2.12–1.73	−2.17 ***	−2.37–1.98	−2.17 ***	−2.37–1.98
White	0.39 ***	0.22–0.56	0.38 ***	0.22–0.55	0.46 ***	0.29–0.63	0.42 ***	0.25–0.60
Financial stress	1.34 ***	1.27–1.40	1.33 ***	1.27–1.40	1.44 ***	1.37–1.51	1.45 ***	1.38–1.52
Enrollment status	0.18	−0.16–0.52	0.17	−0.16–0.51	0.24	−0.10–0.58	0.27	−0.07–0.61
Transfer	−0.05	−0.15–0.06	−0.05	−0.15–0.06	−0.05	−0.15–0.06	−0.06	−0.17–0.04
Parent Education	0.02	−0.03–0.07	0.02	−0.03–0.08	0.02	−0.03–0.07	0.01	−0.04–0.07
Abuse victimization	2.74 ***	2.55–2.94	2.80 ***	2.60–3.00	2.81 ***	2.42–3.20	2.54 ***	2.12–2.95
Constant	4.81 ***	4.08–5.54	4.82 ***	4.09–5.55	4.96 ***	4.23–5.70	5.08 ***	4.34–5.82

*** *p* < 0.001.

**Table 3:** Summary of linear regressions for the association between violence victimization and depressive symptoms.

	B	95% CI	B	95% CI	B	95% CI	B	95% CI
	Any		Emotional		Physical		Sexual	
Age	−0.06 ***	−0.07–0.04	−0.06 ***	−0.08–0.04	−0.06 ***	−0.08–0.04	−0.06 ***	−0.08–0.04
Female	0.35 ***	0.19–0.51	0.36 ***	0.20–0.53	0.57 ***	0.41–0.74	0.53 ***	0.37–0.70
Heterosexual	−2.82 ***	−3.02–2.61	−2.82 ***	−3.02–2.61	−3.10 ***	−3.31–2.89	−3.08 ***	−3.29–2.88
White	−0.12	−0.30–0.06	−0.13	−0.31–0.05	−0.04	−0.22–0.15	−0.08	−0.26–0.11
Financial stress	1.57 ***	1.50–1.65	1.57 ***	1.50–1.65	1.69 ***	1.62–1.76	1.70 ***	1.63–1.78
Enrollment status	0.35	−0.01–0.71	0.35	−0.01–0.71	0.43 *	0.07–0.80	0.46 *	0.10–0.83
Transfer	−0.01	−0.12–0.10	−0.01	−0.12–0.10	−0.01	−0.12–0.11	−0.03	−0.14–0.09
Parent Education	0.02	−0.04–0.07	0.02	−0.04–0.08	0.02	−0.04–0.07	0.01	−0.05–0.06
Abuse victimization	3.09 ***	2.88–3.30	3.14 ***	2.93–3.36	3.05 ***	2.63–3.47	3.05 ***	2.60–3.50
Constant	7.11 ***	6.32–7.89	7.14 ***	6.35–7.92	7.29 ***	6.50–8.09	7.42 ***	6.62–8.21

* *p* < 0.05, *** *p* < 0.001.

**Table 4:** Summary of logistic regressions for the association between violence victimization and suicidal behaviors.

	OR	95% CI	OR	95% CI	OR	95% CI	OR	95% CI
	Any		Emotional		Physical		Sexual	
Age	0.97 ***	0.96–0.98	0.97 ***	0.95–0.98	0.97 ***	0.95–0.98	0.96 ***	0.95–0.98
Gender (Female)	0.80 ***	0.73–0.88	0.81 ***	0.74–0.89	0.89 *	0.81–0.98	0.87 **	0.79–0.96
Heterosexual	0.37 ***	0.34–0.41	0.37 ***	0.34–0.41	0.34 ***	0.31–0.38	0.34 ***	0.31–0.38
Race (White)	1.00	0.90–1.11	1.00	0.90–1.11	1.03	0.93–1.15	1.01	0.91–1.12
Financial stress	1.41 ***	1.35–1.48	1.42 ***	1.36–1.48	1.48 ***	1.42–1.55	1.49 ***	1.43–1.55
Enrollment status	1.27 **	1.07–1.50	1.26 *	1.03–1.54	1.30 *	1.06–1.59	1.32 **	1.08–1.61
Transfer status	1.06 #	1.00–1.14	1.06	0.99–1.13	1.06	0.99–1.13	1.05	0.98–1.12
Parent Education	1.04 **	1.01–1.08	1.04 **	1.01–1.08	1.04 *	1.01–1.07	1.04 *	1.00–1.07
Abuse victimization	2.98 ***	2.71–3.29	2.90 ***	2.62–3.20	2.81 ***	2.37–3.34	2.74 ***	2.27–3.30
Constant	0.12 ***		0.13 ***		0.15 ***		0.16 ***	

# *p* < 0.1, * *p* < 0.05, ** *p* < 0.01, *** *p* < 0.001.
